# The Structural and Functional Capacity of Ruminal and Cecal Microbiota in Growing Cattle Was Unaffected by Dietary Supplementation of Linseed Oil and Nitrate

**DOI:** 10.3389/fmicb.2017.00937

**Published:** 2017-05-24

**Authors:** Milka Popova, Emily McGovern, Matthew S. McCabe, Cécile Martin, Michel Doreau, Marie Arbre, Sarah J. Meale, Diego P. Morgavi, Sinéad M. Waters

**Affiliations:** ^1^UMR1213 Herbivores, Institut National de la Recherche Agronomique, VetAgro Sup, Clermont Université, Université de LyonSaint Genès-Champanelle, France; ^2^Animal and Bioscience Research Department, Animal and Grassland Research and Innovation Centre, TeagascDunsany, County Meath, Ireland

**Keywords:** ruminants, rumen, cecum, methane, linseed, nitrate, microbiota

## Abstract

Microorganisms in the digestive tract of ruminants differ in their functionality and ability to use feed constituents. While cecal microbiota play an important role in post-rumen fermentation of residual substrates undigested in the rumen, limited knowledge exists regarding its structure and function. In this trial we investigated the effect of dietary supplementation with linseed oil and nitrate on methane emissions and on the structure of ruminal and cecal microbiota of growing bulls. Animals were allocated to either a CTL (control) or LINNIT (CTL supplemented with 1.9% linseed and 1.0% nitrates) diet. Methane emissions were measured using the GreenFeed system. Microbial diversity was assessed using amplicon sequencing of microbial genomic DNA. Additionally, total RNA was extracted from ruminal contents and functional *mcrA* and *mtt* genes were targeted in amplicon sequencing approach to explore the diversity of functional gene expression in methanogens. LINNIT had no effect on methane yield (g/kg DMI) even though it decreased methane production by 9% (g/day; *P* < 0.05). *Methanobrevibacter*- and *Methanomassiliicoccaceae*-related OTUs were more abundant in cecum (72 and 24%) compared to rumen (60 and 11%) irrespective of the diet (*P* < 0.05). Feeding LINNIT reduced the relative abundance of *Methanomassiliicoccaceae mcrA* cDNA reads in the rumen. Principal component analysis revealed significant differences in taxonomic composition and abundance of bacterial communities between rumen and cecum. Treatment decreased the relative abundance of a few *Ruminococcaceae* genera, without affecting global bacterial community structure. Our research confirms a high level of heterogeneity in species composition of microbial consortia in the main gastrointestinal compartments where feed is fermented in ruminants. There was a parallel between the lack of effect of LINNIT on ruminal and cecal microbial community structure and functions on one side and methane emission changes on the other. These results suggest that the sequencing strategy used here to study microbial diversity and function accurately reflected the absence of effect on methane phenotypes in bulls treated with linseed plus nitrate.

## Introduction

The complex rumen microbial ecosystem provides the host animal with energy by degrading dietary substrates and producing volatile fatty acids (VFAs; Forbes and France, 1993). Methane production naturally occurs during microbial feed fermentation in the rumen. Similar to ruminal fermentation, in the ruminant's cecum feed digestion is also performed by a specialized consortia of microorganisms. Fermentable substrates arriving in the cecum are different to those in the rumen, which may result in compositional or structural differences in the microbiota of these two compartments. The cecum provides an extra energy source, up to 8.6% of metabolizable energy intake (Siciliano-Jones and Murphy, 1989), which may influence the host animal's metabolism. Also, ~10% of the methane produced by ruminants is derived from microbial activity in the cecum (Murray et al., 1976). Methane production by livestock is a major environmental concern, as it contributes up to 14.5% to anthropogenic greenhouse gas emissions (Gerber et al., 2013). However, while the relationship between ruminal microbes and methane emissions has been extensively studied (Attwood et al., 2011; Creevey et al., 2014; Wallace et al., 2015), information on cecal microbiota structure is scarce. Hence, the characterization of cecal microbiota together with the ruminal would facilitate a greater understanding of the effects of dietary manipulation on microbial and metabolic processes, such as methanogenesis.

Dietary lipids and nitrates have both been identified as effective feeding strategies for enteric methane mitigation (Hristov et al., 2013). However, adding linseed to the diets of beef cattle increases the production costs and regarding dietary nitrates, recommended doses may affect animal health (Doreau et al., 2014). Previous studies from our group showed an additive, as well as a long-term effect of linseed plus nitrate supplemented diets on methane mitigation (Guyader et al., 2015b, 2016). To reduce treatment costs as well as risks for animal health, the associative low-dose supplementation of linseed plus nitrate seems to be an attractive alternative.

Dissolved hydrogen (H_2_) is the limiting substrate of enteric methanogenesis, and manipulating its pool is a well-known lever for mitigating methane emissions (Janssen, 2010). Simultaneous utilization of linseed and nitrate aims to influence both hydrogen production and consumption metabolic pathways. Linseed plus nitrates have toxic effects on hydrogen producing protozoa (Morgavi et al., 2010) and methanogens (Latham et al., 2016), respectively. Additionally, nitrate reduction is a thermodynamically more favorable pathway than the reduction of carbon dioxide to methane. Using stoichiometric calculations, one mole of nitrate should remove one mole of methane (van Zijderveld et al., 2010), though efficiency is always lower than predicted (Leng, 2014) and hydrogen may accumulate in the rumen (Guyader et al., 2015b). Precise antimicrobial mode of action remains nevertheless unclear. Research has shown that linseed supplementation reduces the number of ruminal protozoa (Guyader et al., 2015b; Martin et al., 2016), but this effect is not always observed (Doreau et al., 2009; Benchaar et al., 2012). Feeding linseed oil to dry cows also decreased the abundance of ruminal methanogens (Guyader et al., 2015b). However, in other trials, with decreased methane yield (g/kg dry matter intake), methanogen numbers remained unchanged (Martin et al., 2016). On the other hand, microbial mechanisms of methane reduction when nitrate is fed have received less attention. To our knowledge, there is only one published trial employing 16S RNA amplicon sequencing and showing minor but consistent microbial responses to nitrate supplementation (Veneman et al., 2015). A deeper and more precise description of linseed and/or nitrate induced changes in microbial community structure and activity is still lacking. In a previous study by our group where bulls were fed a high starch diet supplemented with linseed, we observed that the amount of methane produced was associated with methanogen diversity and activity rather than to their number (Popova et al., 2011). This indicates a methanogenic response of the resident methanogens to the supply of their main substrate, hydrogen. Previous findings (Shi et al., 2014) support the utilization of activity-based approaches for studying microbial responses to dietary manipulation. Methanogenic functional *mcrA* gene, coding for an enzyme from the methanogenesis pathway, is a useful molecular marker (Luton et al., 2002), as there are strong correlations between its expression levels in ruminal methanogens and methane yield in sheep, in the absence of significant changes in methanogen community structure or relative abundance (Shi et al., 2014).

The present study aimed to describe and compare the overall microbial community composition in the rumen and cecum ecosystems and to examine the effects of dietary linseed plus nitrate supplementation on the microbial diversity and activity, methane production and fermentation characteristics in growing Charolais bulls. We employed amplicon sequencing of bacterial and archaeal 16S rDNA and *mcrA* cDNA to assess the diversity of bacterial and archaeal community, as well as the diversity of metabolically active archaea. Animal feed intake and live weight gain will be summarized in a companion paper (Doreau et al., under review).

## Materials and methods

The experiment was conducted at the animal facilities of INRA Herbipôle Unit (Saint-Genès Champanelle, France). Procedures on animals were carried out in accordance with the guidelines for animal research of the French Ministry of Agriculture and all other applicable national and European guidelines and regulations for experimentation with animals (see http://www2.vet-lyon.fr/ens/expa/acc_regl.html for details). The protocol was accepted by the Regional Ethics Committee on Animal Experimentation C2EA-02 with reference number 9152-2017030615511441.

### Animals, diet, and experimental design

Sixteen young Charolais bulls, destined for the beef cattle industry, were fed from weaning at 9 months of age up to 14 months a control diet containing 67% wrapped grass silage and 33% concentrate comprising primarily of maize, wheat and rapeseed meal (Table S1). At 14 months of age, the bulls were allotted in two groups balanced by feed intake, body weight, and methane emissions. Animals from the control (CTL) group were fed the same diet; in the treated group (LINNIT), rapeseed meal and a portion of cereals were replaced by extruded linseed and calcium nitrate so that linseed fatty acids and nitrate supply represented 1.9 and 1.0% of dietary DM, respectively. The finishing period lasted at least 7 weeks, as the slaughters at INRA's experimental slaughterhouse staggered over 6 weeks. Methane emissions were measured continuously for 1 month before slaughter using the GreenFeed system (C-lock, Rapid City, SD, USA) as previously described (Arbre et al., 2016). It has been previously estimated that a total of 12 animals are at minimum required to detect a 20% significant decrease in methane emissions measured with the GreenFeed system (Arbre et al., 2016).

### Digesta sample collection

Representative samples of total ruminal and cecal contents were collected after slaughter and immediately snap frozen in liquid nitrogen. Samples were stored at −80°C until molecular analysis. Approximately 150 g of ruminal and cecal contents were used for DM determination (103°C for 24 h); while another sample (~100g) was strained through a polyester monofilament fabric (250 μm mesh aperture) and the filtrate was used for VFA analysis and for protozoa enumeration as previously described (Popova et al., 2013).

### DNA extraction and 16S rDNA amplicon sequencing

Genomic DNA (gDNA) was extracted from each ruminal and cecal sample using bead-beating and on-column purification (Popova et al., 2010). DNA extracts were quantified on a Nanodrop-1000 Spectrophotometer (Thermo Fisher Scientific, France) and run on a FlashGel System (Lonza, Rockland, Inc) to check integrity. Approximately 3 μg of gDNA were sent to Roy J. Carver Biotechnology Center (Urbana, IL61801, USA) for fluidigm amplification and MiSeq Illumina sequencing. Selected primers specifically amplified bacterial and archaeal 16S rDNA (Table S2).

### Total RNA extraction and cDNA synthesis

Ruminal samples were finely ground under liquid nitrogen with a pestle and mortar and stored at −80°C. On average 670 (±9) mg of the frozen powder was used for RNA extraction. RNA was extracted using the RNeasy Plus Mini kit (Qiagen). Total extracted RNA was quantified using a NanoDrop-1000 and quality was tested on a BioAnalyzer 2100 (Agilent Technologies, Inc, France). Traces of DNA were removed through the use of Turbo DNA free kit (Life Technologies) and RNA was further purified and concentrated (RNA Clean & Concentrator kit, Zymo Research, France). Final RNA concentration was set to 50 ng/μl before initiating cDNA synthesis using random primers and High-Capacity cDNA Reverse Transcription kit (Applied Biosystems).

### qPCR quantification of archaeal gene copies in ruminal contents

Gene copies of 16S rDNA for all archaea, methyl co-enzyme M gene (*mcrA*) for all methanogens, and methyltransferase gene (*mtt*) for methylotrophic methanogens were quantified using a qPCR approach. Primers used are summarized in Table S2; reaction assay and temperature cycles for *mcrA* and archaeal 16S rDNA (Popova et al., 2011), and *mtt* (Poulsen et al., 2013) were as described previously. Triplicate qPCR quantification was performed on 20 ng of extracted DNA or 2 μl of cDNA. Amplifications were carried out using SYBR Premix Ex Taq (TaKaRa Bio Inc., Otsu, Japan) on a StepOne system (Applied Biosystems, Courtabeuf, France). Absolute quantification of *mcrA* and 16S rDNA copies involved the use of standard curves that had been prepared with gDNA of *Methanobrevibacter ruminantium* DSM 1093. PCR efficiencies were 95 and 103% for *mcrA* and 16S rDNA, respectively. Expression of the functional *mcrA* and *mtt* genes was assessed using relative quantification by the threshold cycle (C_T_) of the qPCR. Levels of expression were compared in animals fed different diets using the ΔC_T_ (see below) and $2^{-\Delta {\text{C}}_{\text{T}}}$-values (Schmittgen and Livak, 2008).

$$\begin{array}{l} \Delta {\text{C}}_{\text{T }mcrA}={\text{C}}_{\text{T }mcrA}-{\text{C}}_{\text{T }16S\;rDNA}\\ \;\Delta {\text{C}}_{\text{T }mtt}={\text{C}}_{\text{T }mtt}-{\text{C}}_{\text{T }16S\;rDNA} \end{array}$$

### *mcrA* amplicon library preparation

Primers targeting the *mcrA* gene (Poulsen et al., 2013; Table S2) were designed with overhang Illumina adapters. Each 25 μl PCR reaction contained 1X KAPA HiFi HotStart ReadyMix, 0.2 μM of each primer and 2.5 μl of cDNA template. Cycle conditions were 95°C (3 min), then 35 cycles of 95°C (30 s), 55°C (45 s), 72°C (30 s), then a final extension of 72°C (5 min). Amplicons were purified (Min Elute® PCR Purification kit, Qiagen) and used as template in a PCR reaction attaching sequencing indices. The index PCR reaction was produced by mixing 5 μl of both Nextera XT Index Primer1 and Primer2, 25 μl of 2X KAPA HiFi HotStart ReadyMix, 10 μl of ultra-pure molecular biology water and 5 μl of purified amplicons. Cycle conditions were the same as above, except that only eight cycles were performed. Aliquots of 10 μL PCR products were analyzed by electrophoresis on a 2% (w/v) agarose gel to verify the presence and size of the amplicons. Amplicon concentrations were estimated using the Nanodrop-1000. All amplicons were pooled in equivalent quantities in one final library and run on one paired-end MiSeq run.

### Sequence data analysis

The sequence files in FASTQ format were processed using mothur software v.1.33.2 (Schloss et al., 2009), according to the standard operating procedure of Kozich et al. (2013; http://www.mothur.org/wiki/MiSeq SOP, accessed in March 2016).

Amplicon sequencing for the 16S rDNA of bacteria and archaea generated 1,244,917 and 192,515 merged paired end-reads, respectively. Approximately 95% of ruminal and 92% of cecal reads passed the initial quality trimming (mean phred score ≥ 25, length ≥ 350 nt, maximum three ambiguous base-calls, <8 homopolymers). Chimera search using chimera.uchime, implemented in mothur, removed 10% and 7% of the remaining bacterial and archaeal 16S rDNA sequences. For bacteria, on average 37 284 (±3,015) ruminal and 31,810 (±8,751) cecal sequences per sample were used for operational taxonomic units (OTUs) picking. Archaea clustering was performed with 5,330 (±2,280) sequences per ruminal and 3,221 (±1,167) per cecal sample.

*mcrA* cDNA amplicon sequencing generated on average 81,032 (±32,810) merged reads per sample and 81% passed initial quality trimming (mean phred score ≥ 25, length ≥ 470 nt, maximum one ambiguous base-call, <8 homopolymers). After annealing, ~9% of the sequences were identified as chimeric, and removed from downstream analysis. A total of 240 684 sequences (16,045 ± 8,536 on average per sample) were used for taxonomic clustering.

Bacterial and archaeal 16S rDNA were clustered using the average neighbor algorithm at 97% of similarity and taxonomy assigned using, respectively, the greengenes v. 13.5 and the RIM-DB (Seedorf et al., 2014). OTUs for *mcrA* sequences were clustered at 84% sequence divergence (Yang et al., 2014). Sequences were assigned to taxa in mothur with a curated *mcrA* reference database (Yang et al., 2014), where recently published sequences of *mcrA* for *Methanomassiliicoccales* were added.

Greengenes OTU table for bacterial 16S rDNA was used in PICRUSt (phylogenetic investigation of communities by reconstruction of unobserved states; Langille et al., 2013) online version available in the online Galaxy platform (https://huttenhower.sph.harvard.edu/galaxy/) to predict functional genes of classified members on ruminal and cecal bacterial populations.

### Statistical analysis

Changes in methane production, dry matter intake, qPCR quantification and protozoa counts in ruminal digesta were analyzed using an independent 2-group Mann-Whitney test in R. Normal distribution in VFAs concentration in ruminal and cecal contents was confirmed by a Shapiro test and data were further analyzed by one-way ANOVA.

*mcrA* sequencing libraries were prepared for ruminal contents, whereas 16S rDNA libraries were constructed for both ruminal and cecal contents. Raw counts per taxonomic level and OTU tables were used to compute relative abundance and then subjected to square root transformation before two-way ANOVA analysis in R. To minimize the variation created by different sample depths subsampling (resampling 100 times at a depth of 6,419 sequences for *mcrA*, 2,000 for archaeal and 17,000 for bacterial 16S rDNA) was used in our study before beta diversity analysis was performed. Alpha diversity values for ruminal and cecal microbial communities in young bulls fed a CTL or LINNIT diet were obtained using various diversity indices (observed species, Chao estimate, and Shannon and Simpson diversity indices). A transformation-based principal coordinate analysis (PCoA) using the Bray-Curtis distance was used to ordinate 15 ruminal *mcrA* cDNA (one control animal was excluded from sequence analysis because of poor RNA extraction yield), 30 archaeal 16S rDNA (two ruminal content libraries, one from each experimental group, yielded low sequence numbers and were not included in data analysis) and 32 bacterial 16S rDNA animal libraries according to the corresponding OTU abundance data. For statistical analysis of beta diversity, we performed a non-parametric matrix-based ANOVA by using adonis implemented in the vegan package for R. For each dataset beta-diversity, the dissimilarities were further compared using the homogeneity of group dispersions test (Anderson, 2006; R function betadisper in package vegan).

Statistical significance was accepted at *P* < 0.05, and a *P* < 0.10 was considered to indicate a trend.

### Nucleotide sequence accession numbers

Raw sequence data were uploaded to NCBI's Sequence Read Archive database and are accessible as BioProject PRJNA369201.

## Results

### Methane production and fermentation parameters

There was no significant difference in dry matter intake (DMI) between young bulls fed CTL or LINNIT diet (Table 1). Daily methane production (g CH_4_/d) decreased by 9% (*P* < 0.05) in animals receiving the diet supplemented with a combination of linseed plus nitrate (Table 1). However, when methane production was expressed per DMI, no significant differences were observed between the two dietary groups. Diet did not affect VFA concentrations and individual proportions in the rumen or cecum. However, there was a strong digestive compartment effect on VFA concentration profiles (Table 2).

**Table 1:** **Dry matter intake and methane emissions from bulls fed control (CTL) diet (*n* = 8) or diet supplemented with a combination of linseed plus nitrate (LINNIT) (*n* = 8)**.

**Item**	**CTL**	**LINNIT**	**SEM**	**Effect treatment**
DMI	11.6	10.9	0.66	0.274
CH_4_ g/d	275.2	250.9	9.70	0.022
CH_4_ g/kg DMI	24.2	23.7	1.80	0.789

*Values are means of 30 daily measures; data were analyzed using 2-group Mann-Whitney test in R*.

**Table 2:** **Volatile Fatty acids concentrations in rumen and cecum contents of bulls fed control (CTL) diet (*n* = 8) or diet supplemented with a combination of linseed plus nitrate (LINNIT) (*n* = 8)**.

	**CTL**		**LINNIT**		**SEM**	**Effect**		
	**Rumen**	**Cecum**	**Rumen**	**Cecum**		**Treatment**	**DC^a^**	**Treatment × DC**
Total VFAs, mM	128.6a	69.7b	119.6a	67.6b	5.604	0.330	<0.001	0.540
Acetate, mM/mM total VFAs	0.714a	0.763b	0.713a	0.766b	0.004	0.769	<0.001	0.665
Propionate, mM/mM total VFAs	0.156a	0.145ab	0.147ab	0.144b	0.003	0.092	0.027	0.221
Isobutyrate, mM/mM total VFAs	0.008a	0.012b	0.008a	0.012b	0.001	1.000	<0.001	0.955
Butyrate, mM/mM total VFAs	0.097a	0.049b	0.106a	0.046b	0.005	0.533	<0.001	0.261
Isovalerate, mM/mM total VFAs	0.012	0.013	0.012	0.013	0.001	0.812	0.805	0.673
Valerate, mM/mM total VFAs	0.009a	0.019b	0.009a	0.018b	0.001	0.621	<0.001	0.697
Caproate, mM/ mM total VFAs	0.004b	0.000a	0.005*c*	0.000a	0.000	0.005	<0.001	0.022
Dry matter of digesta (%)	11.8a	10.2b	11.9a	10.0b	0.336	0.865	<0.001	0.628

*Values are means of eight individual measures; data were analyzed using one-way ANOVA Different letters in the same row represent significant differences (P < 0.05)*.

a *digestive compartment*.

### Protozoa

Dietary treatment had no effect (*P* > 0.05) on protozoa numbers in the rumen with small Entodiniomorphs (<100 μm) being the more abundant (6.9 × 10^5^ cells/mL ± 9.54 × 10^4^), followed by large Entodiniomorphs (2.6 × 10^4^ ± 7.67 × 10^3^), *Dasytricha* (1.6 × 10^4^ cells/mL ± 5.74 × 10^3^), and *Isotricha* sp. (2.5 × 10^3^ ± 1.55 × 10^3^).

### Methanogenic archaea

Dietary supplementation with linseed plus nitrates had no effect on methanogenic archaea numbers in ruminal contents as both 16S rDNA and *mcrA* copy numbers were similar between CTL and LINNIT animals (Table 3). Archaeal 16S rDNA sequences from ruminal and cecal contents clustered in 343 OTUs at 97% similarity but the 10 most abundant OTUs grouped 99% of all sequences. *Methanobacteriales* represented 70% of the sequences, followed by *Methanomassiliicoccales* (29%). The most abundant OTU was affiliated with the *Methanobrevibacter* genus in both ruminal and cecal contents (Table S3). At the species level (Figure S1), *Methanobrevibacter gottschalkii* and *M. ruminantium* were prelevant in both rumen and cecum, followed by an unclassified species of *Methanomassiliicocaceae Group 9* genus. The abundance of *M. gottschalkii* was, however, higher in cecum, compared to rumen; whereas Group 10 was only detected in ruminal contents. *Methanosphaera sp ISO3-F5* relative abundance in cecum was less than half of that in the rumen. Treatment only affected the relative abundance of *M. ruminantium*, which was decreased by almost 40% in LINNIT animals (Table 4).

**Table 3:** **qPCR quantification of 16S rDNA and *mcrA* copy numbers and *mcrA* and *mtt* expression analysis in rumen contents**.

	**CTL**	**LINNIT**	**SEM**	**Effect treatment**
**DNA**				
16S rDNA log (copies/mL)	3.764	4.162	0.213	0.336
*mcrA* log (copies/mL)	3.418	3.761	0.220	0.463
**RNA**				
ΔC_T_ *mcrA*	6.391	5.319	0.793	0.852
ΔC_T_ *mtt*	12.980	10.244	1.290	0.202
2^−ΔCT *mcrA*^	0.024	0.031	0.007	0.852
2^−CT *mtt*^	0.001	0.003	0.084	0.202
*mcrA* cDNA/*mcrA* DNA	0.166	0.149	0.007	0.106

*Values are means of eight individual measures; data were analyzed using 2-group Mann-Whitney in R*.

**Table 4:** **Methanogenic species relative abundance proportions, based on 16S rDNA reads taxonomic classification, in the rumen and cecum of bulls receiving a control (CTL) or linseed plus nitrate (LINNIT) supplemented diet**.

	**CTL**		**LINNIT**		**SEM**	**Effect**		
	**Rumen**	**Cecum**	**Rumen**	**Cecum**		**Treatment**	**DC^a^**	**Treatment × DC**
*Methanobrevibacter gottschalkii*	0.360	0.504	0.439	0.596	0.046	0.093	0.005	0.888
*Methanobrevibacter ruminantium*	0.198	0.198	0.154	0.085	0.030	0.013	0.260	0.254
unclassified Group9	0.133	0.123	0.113	0.176	0.032	0.651	0.484	0.336
Group10 sp	0.123	–	0.138	–	0.014	0.713	0.000	0.713
*Methanosphaera sp ISO3-F5*	0.044	0.024	0.053	0.013	0.009	0.924	0.005	0.340
Group12 sp	0.036	–	0.028	0.000	0.004	0.505	0.000	0.499
unclassified Methanosphaera	0.033	0.006	0.014	0.009	0.009	0.457	0.136	0.306
Group9 sp	0.021	0.000	0.007	0.000	0.003	0.228	0.015	0.222
unclassified Methanobrevibacter	0.018	0.013	0.016	0.023	0.004	0.413	0.835	0.230
*Methanobrevibacter boviskoreani*	0.013	0.014	0.012	0.018	0.007	0.858	0.604	0.739
*Methanosphaera stadtmanae*	0.008	0.001	0.020	0.003	0.003	0.053	0.001	0.126
Group8 sp	0.005	0.116	0.001	0.067	0.016	0.252	0.001	0.329
*Methanobacterium alkaliphilum*	0.003	–	0.001	0.009	0.002	0.155	0.363	0.033
Group11 sp	0.003	–	0.001	–	0.001	0.225	0.078	0.225
*unclassified_Methanobacteriaceae*	0.000	0.000	0.000	0.000	0.000	0.457	0.136	0.306
Methanobrevibacter oralis	0.000	0.000	0.000	0.000	0.000	0.211	0.289	0.307
unclassified Methanomicrobiaceae	0.000	–	–	–	0.000	0.326	0.326	0.326
unclassified	0.000	–	0.000	–	0.000	0.937	0.169	0.937
*Methanobacterium formicicum*	–	–	0.000	0.000	0.000	0.277	0.388	0.388
*Methanimicrococcus blatticola*	–	–	0.000	–	0.000	0.326	0.326	0.326

a *digestive compartment*.

*Values are means of eight individual measures; data were analyzed using two-way ANOVA in R*.

Shannon and Simpson diversity indices were higher and lower in the rumen, respectively, compared to the cecum (Table S4). However, no treatment effect was observed on any index of diversity.

A total of 280 *mcrA* cDNA OTUs were constructed with the six most abundant OTUs grouping 99% of sequences. These represented the orders *Methanobacteriales* (65%), *Methanomassiliicoccales* (2%), and an unclassified *Euryarchaeota* order (33%). More than 50% of the *mcrA* cDNA reads closely clustered in a *Methanobrevibacter*-affiliated OTU in both control and treated animals (Table 5). Feeding linseed plus nitrate reduced the relative abundance of *Methanomassiliicoccaceae mcrA* cDNA reads (Table 5) in the rumen. Similarly, the Chao1 diversity estimator tended to decrease (−64%, *P* = 0.06) in ruminal contents of linseed plus nitrates fed young bulls (Table S4).

**Table 5:** ***mcrA* cDNA relative abundance proportions in the rumen of bulls receiving a control (CTL) or linseed plus nitrate (LINNIT) supplemented diet; only the six most abundant OTUs (clustering at 97% of similarity) are presented, regrouping 99% of sequences**.

**OTU n°**	**Affiliation (bootstrap values)**	**CTL**	**LINNIT**	**SEM**	**Effect treatment**
1	*Methanobrevibacter* (100)	0.58	0.65	0.065	0.41
2	Unclassified Euryarchaeota (100)	0.18	0.13	0.025	0.16
5	Unclassified Euryarchaeota (100)	0.13	0.09	0.04	0.42
6	uncultured_rumen_archaeon (100)	0.07	0.11	0.03	0.31
4	unclassified Methanomassiliicoccaceae (53)	0.03	0.01	–	<0.001
3	unclassified Methanomicrobiales (95)	0.01	0.01	–	0.24

*Values are means of eight individual measures; data were analyzed using one-way ANOVA in R. Bootstrap values were computed in mothur following 1,000 iterations*.

Analysis of ΔC_T_-values for *mcrA* and *mtt* cDNAs revealed a numeric decrease in expression levels in LINNIT compared to CTL fed young bulls (Table 3).

### Bacteria

In total, 21 bacterial phyla were identified within ruminal and cecal microbiota. Firmicutes and Bacteroidetes were the dominant phyla regardless of digestive compartment or dietary treatment (Table 6), whereas 11 phyla had a mean abundance of <1%; these were Verrucomicrobia, Chloroflexi, Candidate division SR1, Elusimicrobia, Planctomycetes, Saccharibacteria, SHA-109, Synergistetes, Chlamydiae, Deinococcus-Thermus, Parcubacteria. Of the 100 most abundant genera, 92% had different relative abundances between rumen and cecum, and 17 were reduced by dietary supplementation with linseed plus nitrates (Table S5). Treatment had a negative effect on four OTUs from the *Ruminococcaceae* family (*Ruminococcus 2, Ruminococcus gauvreauii* group, *Ruminococcaceae* UCG-005, *Eubacterium coprostanoligenes* group) both in the rumen and cecum. The abundance of other representatives of the *Clostridiales (Anaerovorax, Lachnospiraceae* ND3007 group, *Clostridium sensu stricto 1*) order were increased in the rumen and decreased in the cecum of young bulls fed LINNIT. Five *Bacteroidales* genera (*Prevotellaceae* NK3B31 group, *unclassified Prevotellaceae, unclassified Bacteroidales* S24-7 group, *unclassified Bacteroidales, unclassified Bacteroidales* RF16 group) were decreased in the cecum of treated animals, however, only the first three were also reduced in the rumen.

**Table 6:** **Phylum-level taxonomic composition of the ruminal and cecal bacterial communities in bulls receiving a control (CTL) or linseed plus nitrate (LINNIT) supplemented diet**.

	**CTL**		**LINNIT**		**SEM**	**Effect**		
	**Rumen**	**Cecum**	**Rumen**	**Cecum**		**Treatment**	**DC^a^**	**Treatment × DC**
Firmicutes	0.486	0.638	0.481	0.642	0.005	0.939	<0.001	0.571
Bacteroidetes	0.282	0.241	0.282	0.217	0.007	0.128	<0.001	0.145
Tenericutes	0.040	0.028	0.042	0.033	0.006	0.144	<0.001	0.434
Fibrobacteres	0.039	0.002	0.042	0.001	0.006	0.547	<0.001	0.564
Spirochaetae	0.200	0.120	0.190	0.120	0.008	0.690	<0.001	0.562
Lentisphaerae	0.032	0.019	0.033	0.024	0.007	0.089	<0.001	0.258
Actinobacteria	0.022	0.015	0.020	0.014	0.007	0.459	0.001	0.826
Proteobacteria	0.017	0.014	0.019	0.016	0.006	0.170	0.067	0.982
unclassified_Bacteria	0.014	0.017	0.014	0.018	0.004	0.881	<0.05	0.491
Cyanobacteria	0.012	0.005	0.011	0.010	0.005	<0.05	<0.001	<0.01
Chloroflexi	0.004	0.000	0.005	0.000	0.003	0.289	<0.001	0.757
Candidate_division_SR1	0.003	0.000	0.004	0.000	0.003	0.234	<0.001	0.095
Elusimicrobia	0.003	0.000	0.002	0.000	0.003	0.125	<0.001	0.771
Planctomycetes	0.002	0.000	0.002	0.000	0.003	0.831	<0.001	0.279
SHA-109	0.001	0.000	0.001	0.000	0.002	0.538	<0.001	0.538
Verrucomicrobia	0.001	0.007	0.001	0.009	0.004	0.124	<0.001	0.0439
Synergistetes	0.001	0.000	0.001	0.000	0.001	0.431	<0.001	0.855
Saccharibacteria	0.001	0.000	0.001	0.001	0.003	0.756	<0.01	0.979
Chlamydiae	0.001	0.000	0.004	0.000	0.002	0.091	<0.001	0.914
Deinococcus-Thermus	0.000	0.000	0.000	0.000	0.000	0.326	0.326	0.326
Parcubacteria	0.000	0.000	0.000	0.000	0.000	0.326	0.326	0.326

a *digestive compartment*.

*Values are means of eight individual measures; data were analyzed using two-way ANOVA in R*.

Changes in the relative abundance of potential nitrate-reducing bacterial genera are summarized in Table 7. An increase in LINNIT bulls was observed for *Clostridium sensu stricto 1* and a tendency was observed for *Selenomonas 1*. However, a numerical increase was shown for most other genera, except *Butyrivibrio* and *unclassified Veillonellaceae*.

**Table 7:** **Relative abundance proportions of nitrate-reducing bacterial genera in the rumen and cecum of bulls receiving a control (CTL) or linseed plus nitrate (LINNIT) supplemented diet**.

**Nitrate-reducing genera**	**CTL**		**LINNIT**		**SEM**	**Effect**		
	**Rumen**	**Cecum**	**Rumen**	**Cecum**		**Treatment**	**DC^a^**	**Treatment × DC**
*Selenomonas*	–	0.003	–	–	0.001	0.138	0.138	0.138
*Selenomonas_1*	0.033	0.002	0.039	0.005	0.003	0.083	<0.001	0.646
*Selenomonas_4*	0.003	–	0.006	–	0.001	0.539	<0.05	0.539
*unclassified_Selenomonadales genus*	–	0.007	0.003	0.010	0.002	0.251	<0.01	0.860
*Butyrivibrio*	0.002	0.028	–	0.021	0.002	0.111	<0.001	0.311
*Butyrivibrio_2*	0.129	0.011	0.136	0.015	0.004	0.214	<0.001	0.745
*Clostridium_sensu_stricto_1*	0.002	0.061	0.003	0.073	0.003	<0.05	<0.001	0.083
*Veillonellaceae_UCG.001*	0.033	–	0.038	–	0.003	0.185	<0.001	0.185
*unclassified_Veillonellaceae genus*	0.013	0.003	0.012	0.005	0.003	0.976	<0.001	0.593

*Values are means of eight individual measures; data were analyzed using two-way ANOVA in R*.

Diversity indices of ruminal and cecal bacterial communities were not influenced by dietary treatment, nor by digestive compartment, except for Chao1 which was higher in the cecum of both groups of young bulls (Table S4). However, the Shannon and Simpson diversity indices were higher and lower in the rumen, compared to cecum, respectively.

### Ordination analysis of microbial community structure and activity

**Figure 1:**
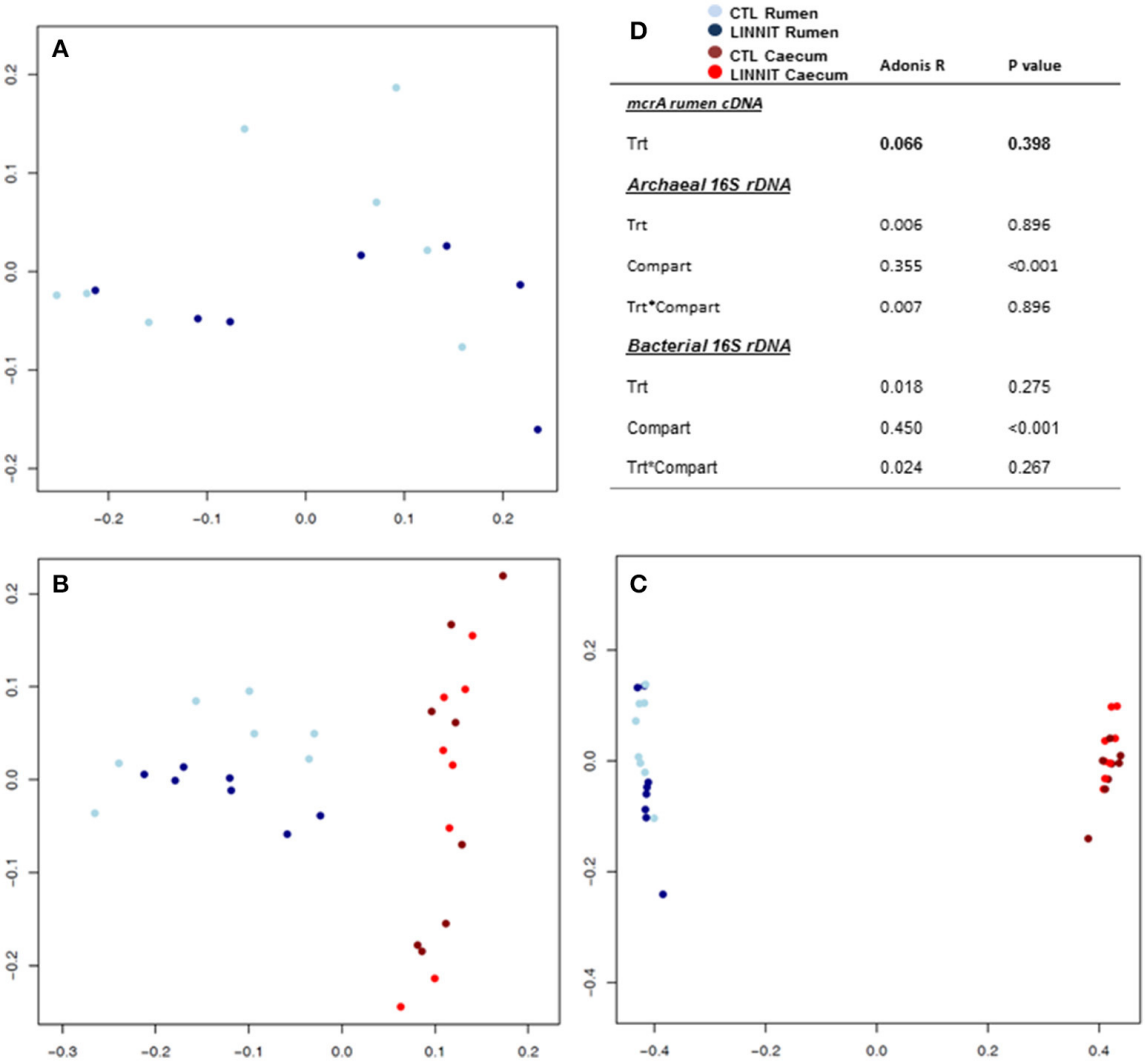
**(A)** Principal coordinate analysis (PCoA) plot of ruminal *mcrA* cDNA reads distribution in CTL and LINNIT animals; **(B)** archaeal; and **(C)** bacterial 16S rRNA gene sequences of microbial communities inhabiting the rumen and cecum of CTL and LINNIT growing bulls. PCoA biplots are based on Bray-Curtis distances *mcrA* and 16S rRNA gene amplicon sequencing data. Statistical comparisons based on the underlying distance matrices are shown in **(D)**.

To analyze the distance between communities within *mcrA* cDNA, archaeal and bacterial 16S rDNA datasets, we used multivariate analysis of variance (adonis) of the bray-curtis distance matrices. This analysis revealed a distinction between rumen and cecum samples, however, no change was observed due to dietary treatment (Figure 1D). Similarly, PCoA of corresponding OTU tables showed that the samples clustered together according to fermentative compartment (Figures 1A–C); some clustering by treatment was apparent for bacterial and archaeal 16S rDNA, though they overlapped resulting in homogenous group dispersions tests (*mcrA P* = 0.871, archaeal 16S rDNA *P* = 0.776, bacterial 16S rDNA *P* = 0.292).

### Predicted molecular functions of ruminal and cecal bacteria

**Figure 2:**
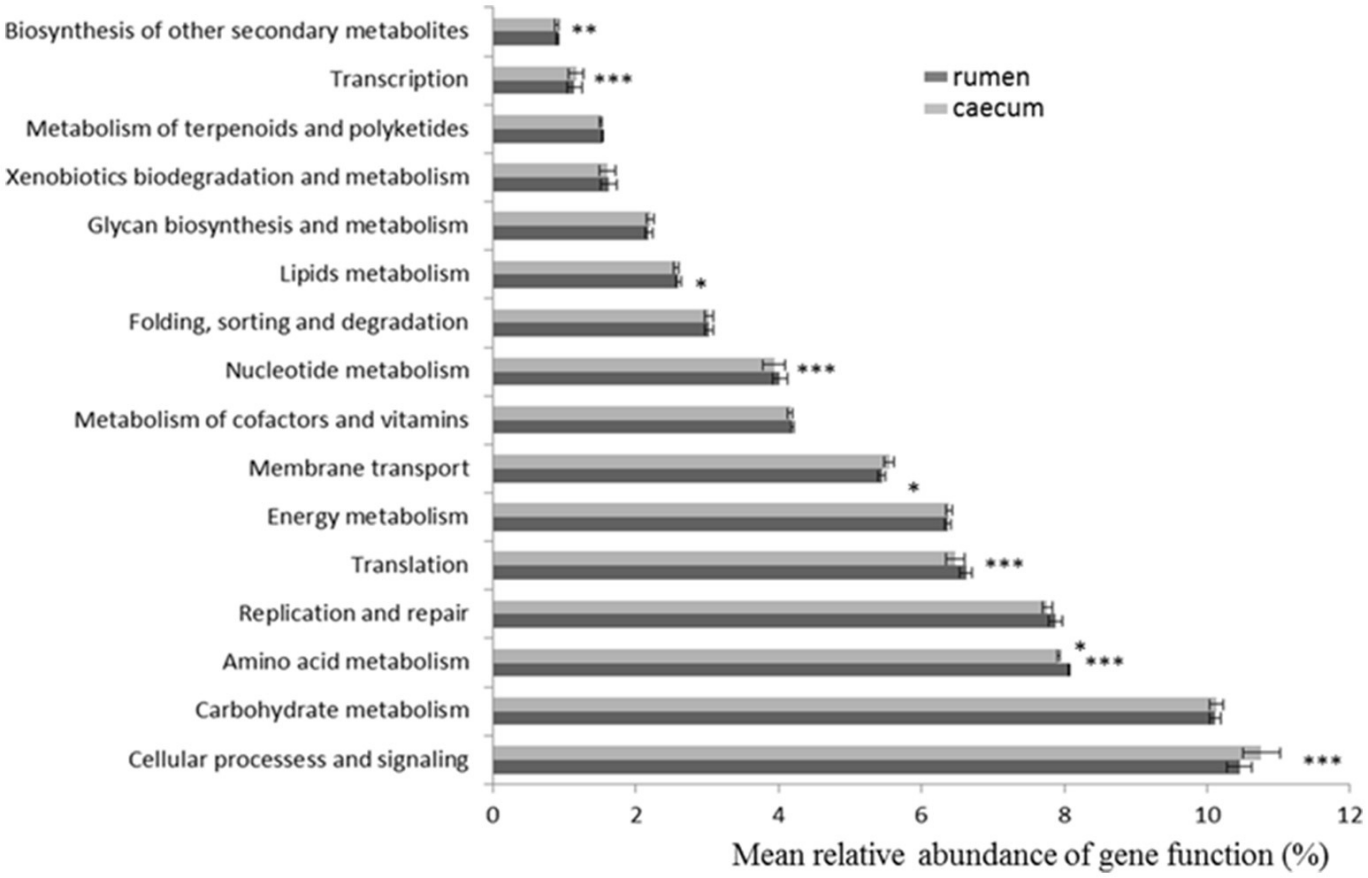
Comparisons of the predominant gene pathways of the bacterial microbiota in rumen and cecum digesta samples predicted by PICRUSt (^***^ <0.001, ^**^ <0.01, ^*^ <0.05).

Using PICRUSt as a predictive exploratory tool, the present study revealed no diet-induced shifts, but a strong effect of the digestive compartment on metagenome contents. Of the 41 KEGG modules (data not shown), the majority of the functional genes belonged to cellular processes and signaling, carbohydrate metabolism, amino acid metabolism, replication and repair, translation, energy metabolism and membrane transport (Table S6). Most were not influenced by diet; moreover, comparing ruminal and cecal samples, irrespective of the diet (Figure 2), further revealed no changes in carbohydrate, energy, terpenoids, polyketides, and xenobiotics metabolism. On the other hand, lipids, nucleotide, and amino acid metabolism were higher in ruminal compared to cecal contents. In more details, “ABC transporters” (PATH:ko02010) were highly represented (more than 3%) in both digestive compartments, followed by genes involved in DNA handling (“DNA repair and recombination proteins” (PATH: ko03400), and “Ribosome” (PATH:ko03010), both accounting for up to 2.3% of ruminal and cecal functional genes). Genes involved in “Methane metabolism” (PATH ko00680) and “Nitrogen metabolism” (PATH ko00910) were more abundant (*P* < 0.05) in the cecum (respectively, 1.24 and 0.69%) compared to the rumen (1.22 and 0.65%), whereas the “Starch and sucrose metabolism” (PATH ko00500) pathway was more represented in the rumen (1.05 vs. 1.03% in the cecum).

## Discussion

We employed high throughput sequencing to assess bacterial and archaeal community profiles in the rumen and cecum of young bulls fed control or linseed plus nitrates supplemented diets. Consistent with the literature (Edwards et al., 2008), Firmicutes and Bacteroidetes were the predominant phyla in both ruminal and cecal libraries. However, differences in abundance were noted for almost all detected phyla. Similarly, ordination analysis of the bacterial community showed a distinct clustering between fermentative compartments. Similar results were observed for bacteria in the gastrointestinal tract of cows and lambs (Michelland et al., 2009; Popova et al., 2013). These results could be explained by the physico-chemical conditions in the two digestive compartments studied, but also by the amount and nature of fermentative substrates available. Diet is the main factor determining relative abundance profiles for most ruminal bacterial species (Henderson et al., 2015). Ruminal microbes degrade more than 90% of dietary plant cell walls (NDF; Huhtanen et al., 2010) and 20–90% of the starch (Moharrery et al., 2014). Feed resources entering the cecum are thus mainly composed of recalcitrant carbohydrates. Nutrient availability has been shown to have a significant selective pressure on the biodiversity of microorganisms in a community (Mello et al., 2016) supporting the lower diversity we observed in the cecum, compared to the ruminal bacterial community. Other factors such as, host genetics, host-derived nutrients, or immune system, may also have a stronger influence on shaping cecal microbial community than for the rumen.

PICRUSt predictions at KEGG level 3 detected many pathways related to metabolism. Among them, ABC transporters, known to mediate the uptake of nutrients (Higgins, 2001) were the most abundant. As previously mentioned, only limited quantities of starch reach the cecum which explain the reduced abundance of genes involved in starch and carbohydrate metabolism in this secondary fermentative compartment, compared to the rumen. Similarly, enhanced nucleotide, amino-acid, and lipid functions were described in ruminal contents. These results are comparable to previously published data in the gastro-intestinal tract of dairy cows (Mao et al., 2015). PICRUSt's predictive metagenome profiling is a cost-effective way to reveal the functional potential of a community. It is noted, however, that inferring functions based on 16S rRNA information has its limitations if reference genomes are not available (Almeida et al., 2016). For the ruminant gastrointestinal tract, only a limited number of bacteria have their genomes sequenced (Creevey et al., 2014), and PICRUSt results should thus be interpreted with caution.

*Methanobrevibacter* (*Methanobacteriaceae* family) and members of *Methanomassiliicoccaceae* were the most abundant archaeal taxa in both the ruminal and cecal libraries constructed with 16S rDNA sequences. *Methanobrevibacter*- and *Methanomassiliicoccaceae*-related sequences are frequently reported in ruminal contents (St-Pierre et al., 2015); the prevalence of *Methanobrevibacter* in cecal contents was reported in growing lambs (Popova et al., 2013) and calves (Zhou et al., 2014). The high abundance *Methanomassiliicoccaceae*-related OTU (24%) in ruminant's cecum is a novel and interesting finding supporting the occurrence of methyl-dependent methanogenesis in the cecum. Shannon and Simpson indices suggested that a more diverse methanogen community exists in the rumen, compared to the cecum. This is consistent will the consensus that diet affects methanogen community structure in the rumen (Henderson et al., 2015; Seedorf et al., 2015). As discussed above, the diversity of nutrients entering the rumen determines the bacterial community composition and thus, the variety of fermentation pathways and microbial interactions. Methanogenic archaea consume final products of fermentation: hydrogen, acetate, or methyl compounds. So it is not surprising that bacterial and archaeal communities were more diverse in the rumen. On the other hand, methanogenesis from acetate is limited in the rumen because the rate of passage of ruminal contents is greater than the growth rate of acetate-utilizing methanogens (Janssen and Kirs, 2008). The relative abundances of *M. stadtmanae, M*. ISO3-F5, as well as unclassified *Methanosphaera* were higher in ruminal contents, where the passage rate is lower (Campling and Freer, 1960), compared to cecum. However, these methanogens remained a minority both in rumen and cecum probably due to substrate competition with the methyl-consuming *Methanomassiliicoccales*-related species; indeed *Methanosphaera* require acetate for growth, but use methanol for methanogenesis.

Daily methane production decreased by 9% in animals fed a diet supplemented with a combination of linseed and nitrate, compared to young bulls fed the control diet. While there was a numerical decrease of 6% in DMI due to supplementation, methane emissions expressed as grams per kilogram of DMI were similar for the CTL and LINNIT groups. Nitrate and linseed oil are both well-known to have a negative effect on methane production in the rumen (Hristov et al., 2013). However, methane yield (g/kg DMI) reduction achieved in this trial was less dramatic compared to recently published reports, e.g., 17% (Troy et al., 2015), 22 and 16% (Veneman et al., 2015) and 22% (Guyader et al., 2015b) with nitrate alone, 17% (Guyader et al., 2015b), 15% (Martin et al., 2016) with linseed alone. The basal diet has also been shown to influence the methane-mitigating effect of nitrate supplementation (Troy et al., 2015). However, the lack of effect was only reported for high concentrate diets, which is not the case in our study. In trials cited above, linseed plus nitrate were supplemented in higher amounts, ~2.2% of DM for nitrate and 2.6% of DM for linseed fatty acids. However, a recent study from our group demonstrated an additive methane-mitigating effect of linseed oil and nitrate when fed simultaneously to non-lactating cows of 32% (Guyader et al., 2015b) and 30% (Guyader et al., 2016). These results led us to study in the current trial, the mitigation effect obtained with a lower dose of each supplement (1.9% for linseed fatty acids and 1% for nitrates) which could be more easily accepted for a large-scale use in commercial farms (Doreau et al., 2014). This dual-supplementation was expected to result in a decrease in methane yield, due to the previous demonstration of a linear relationship between reduced methane emissions and increased levels of linseed (Martin et al., 2016) or nitrate (Lee and Beauchemin, 2014). It is apparent that the dose supplied to young bulls in the current study was insufficient to illicit the same responses observed previously with the supplementation of these additives, either individually or in combination. However, the doses at which they were required to elicit such effects may render them cost-prohibitive and detrimental to animal health, factors which must be addressed before such a dietary strategy could be implemented in the industry.

The limited effect of supplementation on methane emissions was consistent with an absence of change in ruminal VFA concentration profiles between young bulls fed CTL or LINNIT diet. In addition, the protozoa population was not affected by linseed plus nitrate supplementation. Effects of nitrate on ruminal protozoa are contrasting. Asanuma et al. (2015) reported 86% reduction in protozoa abundance in goats receiving potassium nitrate, but their numbers were not affected in lambs (van Zijderveld et al., 2010) or cows (Guyader et al., 2015a). Similarly contrasting results were reported with the addition of linseed oil, some authors observing a linear reduction in protozoa numbers with increasing amounts of linseed (Martin et al., 2016), whereas others report no change even with high amounts of added fatty acids (Benchaar et al., 2012).

PCoA analysis showed some clustering by diet for bacterial and archaeal 16S rDNA samples though they overlap. This is comparable to the results reported by Veneman et al. (2015), who also reported only limited effects of linseed or nitrates supplementation on archaeal and bacterial 16S rDNA libraries. However, effect of dietary treatment on methanogens in both compartments was limited. Accordingly, the abundance of ruminal methanogens was unaffected by increasing linseed supply (Martin et al., 2016). In contrast, nitrite inhibited methanogen growth *in vitro* (Iwamoto et al., 2002) and their numbers were reduced *in vivo* when nitrates were fed to sheep (van Zijderveld et al., 2010). Veneman et al. (2015) also observed a deleterious effect on archaeal numbers with nitrate, but only on the solid fraction of ruminal contents. The niche-specific sensitivity of methanogens to dietary linseed plus nitrates supply is consistent with our methanogen functional sequencing results showing a decrease in *mcrA* transcription for *Methanomassiliicoccaceae* in the rumen of LINNIT young bulls. This taxonomic group uses methylamines as substrates for methanogenesis (Paul et al., 2012), whereas most of the other ruminal methanogens are hydrogenotrophic (Janssen, 2010). Our results are in agreement with previous studies, where *Methanomassiliicoccaceae* numbers were reduced upon dietary supplementation with rapeseed oil in lactating cows (Poulsen et al., 2013). Furthermore, we also observed a numerical decrease in *mcrA* and *mtt* expression levels. It should be noted however, that *mcrA* primers used in sequencing library construction and qPCR assays were not the same. Indeed, sequencing primers were more degenerated assuring better coverage of the *Methanomassiliicoccaceae* group (Poulsen et al., 2013).

The effect of linseed plus nitrates supplementation was less evident on bacteria, which is consistent with analysis of Veneman et al. (2015) that showed small changes in ruminal community structure due to linseed or nitrates treatment. Among the 100 most abundant genera, only 17 were affected by dietary treatment. Polyunsaturated fatty acids are known to have a toxic effect on some ruminal bacteria, especially cellulolytic ones (Maia et al., 2007), however, we did not observe a reduction in the numbers of the three main cellulolytic bacteria (*Fibrobacter succinogenes, Ruminococcus albus, Ruminococcus flavefaciens*). *In vitro*, nitrate inhibited growth of the same three species; *in vivo* their numbers were reduced in the rumen of goats (Asanuma et al., 2015), but increased in steers (Zhao et al., 2015) fed nitrates. *Ruminococcus* species adapt to nitrates, whereas methanogens and *F. succinogenes* do not (Zhou et al., 2012). However, in our study, the relative abundance of four *Ruminococcus* species decreased with the addition of linseed plus nitrates, but we observed no change in *Fibrobacter* species abundance. Studying digestibility and cellulolytic microbes was not the aim here. The effect of treatment on this microbial population should be explored further in other trials and conditions, as their function is fundamental for optimal ruminal fermentation. Accurate quantification methods, flow cytometry, or qPCR could be considered and coupled with amplicon DNA sequencing and transcriptomic-based studies.

*Selenomonas ruminantium, Veillonella parvula*, and *Wolinella succinogenes* have all been confirmed to be active in nitrate reduction (Iwamoto et al., 2002). While *in vitro, W. succinogenes* possessed higher activity (Iwamoto et al., 2002), *in vivo S. ruminantium* is the most abundant (Asanuma et al., 2015; Veneman et al., 2015). Accordingly, in our study we only detected sequences affiliated to the genus *Selenomonas*. Their relative abundance was low and they were not present in all animals which explains the numerical but not significant increase in the presence of nitrate. Sequencing results of Veneman et al. (2015) also showed no change in *Selenomonas* abundance which contrasted to qPCR quantification data of Zhao et al. (2015). Induction of nitrate and nitrite reduction pathways in other fermentative bacteria such as *Butyrivibrio* or *Clostridium* could also be expected, though they are present in low abundance (Latham et al., 2016). It is also likely, that all ruminal nitrate reducers have not yet been identified; hence it would be interesting to perform a thorough search of available ruminal metagenomes for genes involved in nitrate and nitrite reduction.

Results from our study revealed that the rumen and cecum of growing bulls harbor dissimilar microbial communities but have comparable metabolic functions. This is an interesting finding, as the cecum plays an important role in post-ruminal fermentation of feed. Thus, better knowledge of microbial assemblages across the gastrointestinal tract of ruminants should allow the identification of novel strategies to modulate their functions. Secondly, linseed plus nitrate dietary supply had a limited effect on methane emission and fermentation parameters, probably because animals were administered lower doses than those previously published, in an attempt to fit in with practical use in commercial farms. The absence of effect on methane yield and VFA profiles was corroborated by the absence of major changes in the structure and functionality of methanogenic and bacterial communities in the rumen and cecum of young Charolais bulls. The sequencing strategy used in this study accurately identified the absence of changes in methane emissions in growing bulls.

## Author contributions

Conception or design of the work: MP, CM, MD, DM, SW. Data collection: MP, MA, SM, EM. Data analysis and interpretation: MP, MA, EM, MM. Drafting the article: MP. Critical revision of the article: MP, MM, CM, MD, DM, SW. Final approval of the version to be published: MP, EM, MM, CM, MD, MA, SM, DM, SW.

### Conflict of interest statement

The authors declare that the research was conducted in the absence of any commercial or financial relationships that could be construed as a potential conflict of interest.
