# Supplementary material for: The Structural and Functional Capacity of Ruminal and Cecal Microbiota in Growing Cattle Was Unaffected by Dietary Supplementation of Linseed Oil and Nitrate
Source: Front Microbiol. 2017 May 24;8:937. doi: 10.3389/fmicb.2017.00937 (PMC5442214; doi:10.3389/fmicb.2017.00937)
Supplement: Supplementary file 1 [file Table1.DOCX]

Table S1 : Chemical composition of experimental diets

|  | CTL diet | LINNIT diet |
| --- | --- | --- |
| Organic matter, g/kg DM | 899 | 894 |
| Crude protein, g/kg DM | 171 | 167 |
| NDF, g/kg DM | 462 | 472 |
| ADF, g/kg DM | 250 | 253 |
| Starch, g/kg DM | 95 | 97 |
| Fatty acids, g/kg DM | 13 | 33 |
