# Supplementary material for: The Structural and Functional Capacity of Ruminal and Cecal Microbiota in Growing Cattle Was Unaffected by Dietary Supplementation of Linseed Oil and Nitrate
Source: Front Microbiol. 2017 May 24;8:937. doi: 10.3389/fmicb.2017.00937 (PMC5442214; doi:10.3389/fmicb.2017.00937)
Supplement: Supplementary file 2 [file Table2.DOCX]

Table S2 : Sequencing and qPCR primers used in this study

| **Target** | **Primer name** | **Primer sequence** | **Usage** | **Size of amplicon (nt)** | **Reference** |
| --- | --- | --- | --- | --- | --- |
| Bacteria 16S rDNA | V4_515F_New  V4_806R_New | 5'-GTGYCAGCMGCCGCGGTAA  5'-GGACTACNVGGGTWTCTAAT | Sequencing | 292 | ([Caporaso et al 2012](#_ENREF_1)) |
| Archaea 16S rDNA | Arch349F  Arch806R | 5'-GYGCASCAGKCGMGAAW  5'-GGACTACVSGGGTATCTAAT | Sequencing | 457 | ([Takai and Horikoshi 2000](#_ENREF_5)) |
| mcrA all methanogens | Mlas-mod  mcrA-rev | 5’-GGY GGT GTM GGD TTC ACM CAR TA  5’-CGT TCA TBG CGT AGT TVG GRT AGT | Sequencing | 460 | ([Poulsen et al 2013](#_ENREF_4)) |
| Archaea 16S rDNA | qArch16S-896-915F  Arch1406-1389r | AGGAATTGGCGGGGGAGCAC  ACGGGCGGTGTGTGCAAG | qPCR | 491 | ([Ohene-Adjei et al 2007](#_ENREF_3)) |
| mcrA all methanogens | qmcrA-f  qmcrA-r | TTCGGTGGATCDCARAGRGC  GBARGTCGWAWCCGTAGAATCC | qPCR | 140 | ([Denman et al 2007](#_ENREF_2)) |
| TMA-methyltransferase gene of *Methanomassiliicoccales* | mtt For  mtt Rev | GYR CTT CMA CCA CAT CGA CC  CRG CCA TBG CCA TGG ACA G | qPCR | 160 | ([Poulsen et al 2013](#_ENREF_4)) |
