# Supplementary material for: The Structural and Functional Capacity of Ruminal and Cecal Microbiota in Growing Cattle Was Unaffected by Dietary Supplementation of Linseed Oil and Nitrate
Source: Front Microbiol. 2017 May 24;8:937. doi: 10.3389/fmicb.2017.00937 (PMC5442214; doi:10.3389/fmicb.2017.00937)
Supplement: Supplementary file 3 [file Table3.DOCX]

**Table S3 :** Archaeal 16S rDNA OTUs relative abundance in the rumen and caecum of bulls receiving a control (CTL) or linseed plus nitrate (LINNIT) supplemented diet; only the ten most abundant OTUs (clustering at 97% of similarity) are presented, regrouping 99% of sequences.

| **OTU n°** | **Affiliation (bootstrap values)** | **CTL** | | **LINNIT** | | **SEM** | **Effect** | | |
| --- | --- | --- | --- | --- | --- | --- | --- | --- | --- |
|  |  | **rumen** | **caecum** | **rumen** | **caecum** |  | **Treatment** | **DC ^1^** | **Treatment x DC** |
| 1 | *Methanobrevibacter* (100) | 0.59 | 0.72 | 0.61 | 0.72 | 0.02 | 0.814 | <0.01 | 0.749 |
| 2 | Unclassified Methanomassiliicoccaceae (71) | 0.13 | 0.24 | 0.09 | 0.24 | 0.02 | 0.623 | <0.01 | 0.298 |
| 3 | *Group 10* (100) | 0.12 | - | 0.14 | - | 0.02 | 0.784 | < 0.001 | 0.784 |
| 5 | *Methanosphaera* (100) | 0.05 | 0.01 | 0.05 | 0.03 | 0 | 0.66 | <0.01 | 0.503 |
| 4 | *Methanosphaera* (100) | 0.03 | 0.02 | 0.04 | 3.4 x10^-3^ | 0.01 | 0. 669 | < 0.001 | 0.091 |
| 6 | *Group 12* (100) | 0.04 | - | 0.03 | 5.8 x10^-5^ | - | 0.791 | < 0.001 | 0.703 |
| 7 | *Group 10* (98) | 0.02 | - | 0.03 | - | - | 0.538 | <0.001 | 0.538 |
| 10 | *Group 8* (100) | 0.01 | - | 1.4 x10^-3^ | 5.8 x10^-5^ | - | 0.264 | <0.01 | 0.181 |
| 8 | *Group 9* (100) | 4.1 x10^-3^ | - | 5.3 x10^-4^ | - | - | 0.751 | <0.05 | 0.751 |
| 9 | *Group 11* (100) | - | 0.01 | - | 4.8 x10^-3^ | - | 0.15 | <0.05 | 0.15 |
|  | ^1^ digestive compartment |  |  |  |  |  |  |  |  |
