# Supplementary material for: The Structural and Functional Capacity of Ruminal and Cecal Microbiota in Growing Cattle Was Unaffected by Dietary Supplementation of Linseed Oil and Nitrate
Source: Front Microbiol. 2017 May 24;8:937. doi: 10.3389/fmicb.2017.00937 (PMC5442214; doi:10.3389/fmicb.2017.00937)
Supplement: Supplementary file 4 [file Table4.docx]

Table S4 : Alpha diversity in mcrA cDNA, archaeal 16S rDNA and bacterial 16S rDNA sequencing libraries.

|  | cDNA *mcrA* | | | | archaeal 16S rDNA | | | | | | | | bacterial 16S rDNA | | | | | | | |
| --- | --- | --- | --- | --- | --- | --- | --- | --- | --- | --- | --- | --- | --- | --- | --- | --- | --- | --- | --- | --- |
|  | rumen | | | | rumen | | caecum | | SEM | P value | | | rumen | | caecum | | SEM | P value | | |
|  | CTL | LINNIT | SEM | P value | CTL | LINNIT | CTL | LINNIT |  | Trt | Compart | Trt*Compart | CTL | LINNIT | CTL | LINNIT |  | Trt | Compart | Trt*Compart |
| Number of sequences | 13347 | 19129 | 3040 | 0.189 | 6030 | 6061 | 3490 | 2953 | 412 | 0.534 | <0.001 | 0.517 | 26780 | 27787 | 34394 | 29226 | 2011 | 0.371 | 0.058 | 0.188 |
| Coverage | 0.99 | 0.99 | 1.4 _x_10^-4^ | 0.152 | 0.99 | 0.99 | 0.99 | 0.99 | 3.3 _x_ 10^-4^ | 0.359 | <0.05 | 0.406 | 0.84 | 0.84 | 0.84 | 0.82 | 0.006 | 0.421 | 0.210 | 0.597 |
| Shannon | 1.12 | 0.99 | 0.119 | 0.397 | 1.36 | 1.29 | 0.66 | 0.68 | 0.056 | 0.756 | <0.001 | 0.453 | 7.13 | 7.15 | 7.15 | 7.26 | 0.052 | 0.270 | 0.228 | 0.478 |
| Simpson | 0.503 | 0.442 | 0.066 | 0.397 | 0.37 | 0.40 | 0.63 | 0.61 | 0.03 | 0.815 | <0.001 | 0.585 | 3.7 _x_ 10^-3^ | 3.6 _x_ 10^-3^ | 3.3 _x_ 10^-3^ | 3 _x_ 10^-3^ | 3.2 _x_ 10^-4^ | 0.591 | 0.136 | 0.839 |
| Chao | 320 | 115 | 49.58 | 0.064 | 116.5 | 67.9 | 53.3 | 58.3 | 19.51 | 0.326 | 0.081 | 0.193 | 10311 | 10306 | 11179 | 11774 | 522.8 | 0.599 | <0.05 | 0.594 |
