# Supplementary material for: The Structural and Functional Capacity of Ruminal and Cecal Microbiota in Growing Cattle Was Unaffected by Dietary Supplementation of Linseed Oil and Nitrate
Source: Front Microbiol. 2017 May 24;8:937. doi: 10.3389/fmicb.2017.00937 (PMC5442214; doi:10.3389/fmicb.2017.00937)
Supplement: Supplementary file 5 [file Table5.docx]

| Table S5 Genus-level taxonomic composition of the ruminal and caecal bacterial communities in bulls fed control (CTL) diet and diet supplemented with linseed and nitrates (LINNIT). Only the 100 most abundant genera are shown. | | | | | | | |
| --- | --- | --- | --- | --- | --- | --- | --- |
|  |  |  |  |  |  |  |  |
|  | **CTL** | | **LINNIT** | | **Effect** | | |
|  | **rumen** | **caecum** | **rumen** | **caecum** | **Treatment** | **DC ^1^** | **Treatment x DC ^1^** |
| Ruminococcaceae_UCG-010 | 1.30E-02 | 1.40E-01 | 1.30E-02 | 1.50E-01 | 0.691 | <0.001 | 0.568 |
| unclassified_Lachnospiraceae | 6.00E-02 | 8.50E-02 | 6.50E-02 | 8.40E-02 | 0.641 | <0.001 | 0.594 |
| Rikenellaceae_RC9_gut_group | 6.20E-02 | 5.90E-02 | 6.40E-02 | 5.20E-02 | 0.408 | <0.05 | 0.153 |
| Christensenellaceae_R-7_group | 7.90E-02 | 3.50E-02 | 7.70E-02 | 4.10E-02 | 0.396 | <0.001 | 0.135 |
| Prevotella_1 | 6.80E-02 | 1.10E-03 | 6.70E-02 | 8.30E-04 | 0.651 | <0.001 | 0.861 |
| unclassified_Ruminococcaceae | 1.40E-02 | 5.10E-02 | 1.30E-02 | 4.70E-02 | 0.272 | <0.001 | 0.536 |
| unclassified_Mollicutes_RF10 | 3.40E-02 | 2.10E-02 | 3.60E-02 | 2.60E-02 | 0.149 | <0.001 | 0.509 |
| **Ruminococcaceae_UCG-005** | **4.50E-03** | **4.90E-02** | **3.60E-03** | **4.50E-02** | **<0.05** | **<0.001** | **0.77** |
| unclassified_Bacteroidales_BS11_gut_group | 5.20E-02 | 2.90E-03 | 5.20E-02 | 2.70E-03 | 0.865 | <0.001 | 0.971 |
| unclassified_Clostridiales | 1.50E-02 | 3.30E-02 | 1.40E-02 | 3.40E-02 | 0.868 | <0.001 | 0.376 |
| Treponema_2 | 3.50E-02 | 1.30E-02 | 3.30E-02 | 1.30E-02 | 0.673 | <0.001 | 0.527 |
| Fibrobacter | 3.90E-02 | 1.20E-03 | 4.20E-02 | 1.10E-03 | 0.466 | <0.001 | 0.649 |
| **Eubacterium_coprostanoligenes_group** | **8.30E-03** | **3.00E-02** | **7.90E-03** | **2.60E-02** | **<0.05** | **<0.001** | **0.124** |
| unclassified_Lentisphaerae_RFP12_gut_group | 2.50E-02 | 1.00E-02 | 2.40E-02 | 1.40E-02 | 0.162 | <0.001 | 0.079 |
| unclassified_Bacteria | 1.40E-02 | 1.70E-02 | 1.40E-02 | 1.80E-02 | 0.881 | <0.05 | 0.491 |
| Bacteroides | 9.00E-04 | 2.90E-02 | 1.10E-03 | 2.70E-02 | 0.657 | <0.001 | 0.169 |
| Ruminococcaceae_UCG-014 | 1.80E-02 | 1.10E-02 | 1.80E-02 | 1.30E-02 | 0.535 | <0.001 | 0.368 |
| Prevotellaceae_UCG-003 | 1.40E-02 | 1.70E-02 | 1.40E-02 | 1.30E-02 | 0.069 | 0.499 | 0.075 |
| **unclassified_Bacteroidales_RF16_group** | **7.80E-03** | **2.00E-02** | **9.40E-03** | **1.60E-02** | **0.37** | **<0.001** | **<0.05** |
| Alistipes | 1.70E-04 | 2.50E-02 | 1.40E-04 | 2.20E-02 | 0.051 | <0.001 | 0.221 |
| **unclassified_Bacteroidales** | **7.00E-03** | **1.70E-02** | **8.20E-03** | **1.50E-02** | **0.705** | **<0.001** | **<0.05** |
| unclassified_Clostridiales_vadinBB60_group | 2.00E-03 | 2.10E-02 | 2.10E-03 | 2.20E-02 | 0.396 | <0.001 | 0.689 |
| Ruminococcus_1 | 1.90E-02 | 3.10E-03 | 1.90E-02 | 3.60E-03 | 0.619 | <0.001 | 0.693 |
| Ruminococcaceae_NK4A214_group | 1.60E-02 | 5.70E-03 | 1.60E-02 | 6.30E-03 | 0.523 | <0.001 | 0.91 |
| Saccharofermentans | 2.00E-02 | 1.80E-03 | 2.00E-02 | 1.70E-03 | 0.934 | <0.001 | 0.999 |
| **unclassified_Prevotellaceae** | **1.20E-02** | **9.10E-03** | **9.50E-03** | **8.80E-03** | **<0.05** | **<0.05** | **0.097** |
| Acetitomaculum | 1.50E-02 | 6.80E-03 | 1.10E-02 | 7.00E-03 | 0.182 | <0.001 | 0.204 |
| **unclassified_Bacteroidales_S24-7_group** | **1.80E-02** | **4.40E-03** | **1.50E-02** | **3.00E-03** | **<0.05** | **<0.001** | **0.838** |
| **unclassified_Gastranaerophilales** | **1.10E-02** | **4.70E-03** | **1.10E-02** | **9.40E-03** | **<0.05** | **<0.001** | **<0.01** |
| Butyrivibrio_2 | 1.70E-02 | 1.80E-04 | 1.90E-02 | 2.80E-04 | 0.214 | <0.001 | 0.745 |
| Lachnospiraceae_NK3A20_group | 1.50E-02 | 3.00E-03 | 1.40E-02 | 3.00E-03 | 0.472 | <0.001 | 0.667 |
| Phocaeicola | 3.00E-04 | 1.50E-02 | 1.60E-04 | 1.50E-02 | 0.313 | <0.001 | 0.452 |
| Ruminiclostridium_5 | 7.10E-03 | 8.70E-03 | 4.50E-03 | 9.30E-03 | 0.226 | <0.001 | 0.077 |
| Prevotellaceae_UCG-001 | 1.20E-02 | 2.40E-03 | 1.10E-02 | 2.40E-03 | 0.145 | <0.001 | 0.194 |
| **Ruminococcus_2** | **6.90E-03** | **9.00E-03** | **3.60E-03** | **5.70E-03** | **<0.01** | **0.059** | **0.956** |
| Victivallis | 2.60E-03 | 8.00E-03 | 3.50E-03 | 9.50E-03 | 0.132 | <0.001 | 0.971 |
| Erysipelotrichaceae_UCG-004 | 8.70E-03 | 3.70E-03 | 8.90E-03 | 3.40E-03 | 0.883 | <0.001 | 0.664 |
| Ruminococcaceae_UCG-013 | 5.50E-04 | 1.10E-02 | 9.10E-04 | 9.80E-03 | 0.872 | <0.001 | 0.089 |
| unclassified_Bacteroidetes | 5.80E-03 | 4.10E-03 | 7.50E-03 | 5.10E-03 | 0.056 | <0.01 | 0.758 |
| Family_XIII_AD3011_group | 3.70E-03 | 6.30E-03 | 3.80E-03 | 5.50E-03 | 0.291 | <0.001 | 0.148 |
| unclassified_Erysipelotrichaceae | 4.90E-03 | 5.40E-03 | 4.40E-03 | 4.90E-03 | 0.234 | 0.342 | 0.832 |
| Eubacterium_ruminantium_group | 1.00E-02 | 1.30E-04 | 9.00E-03 | 1.70E-04 | 0.568 | <0.001 | 0.556 |
| Atopobium | 6.20E-03 | 3.60E-03 | 4.30E-03 | 3.90E-03 | 0.176 | <0.01 | 0.112 |
| unclassified_Coriobacteriaceae | 5.20E-03 | 3.40E-03 | 4.90E-03 | 4.20E-03 | 0.539 | <0.05 | 0.309 |
| unclassified_Rikenellaceae | 9.40E-04 | 7.50E-03 | 1.30E-03 | 7.00E-03 | 0.746 | <0.001 | 0.209 |
| Lachnospiraceae_XPB1014_group | 7.90E-03 | 7.30E-05 | 9.40E-03 | 1.10E-04 | 0.169 | <0.001 | 0.167 |
| unclassified_Firmicutes | 2.10E-03 | 5.60E-03 | 1.80E-03 | 6.10E-03 | 0.995 | <0.001 | 0.148 |
| Akkermansia | 0.00E+00 | 6.60E-03 | 6.30E-05 | 8.30E-03 | 0.059 | <0.001 | 0.371 |
| Prevotellaceae_UCG-004 | 2.80E-03 | 5.10E-03 | 2.60E-03 | 5.00E-03 | 0.68 | <0.001 | 0.687 |
| Succiniclasticum | 7.20E-03 | 2.10E-04 | 7.90E-03 | 1.10E-04 | 0.909 | <0.001 | 0.266 |
| Lachnospiraceae_NK4A136_group | 2.50E-03 | 4.30E-03 | 2.90E-03 | 4.50E-03 | 0.28 | <0.001 | 0.58 |
| Senegalimassilia | 5.20E-03 | 1.40E-03 | 5.90E-03 | 2.10E-03 | 0.183 | <0.001 | 0.811 |
| Lachnospiraceae_AC2044_group | 6.40E-03 | 4.20E-04 | 6.90E-03 | 5.60E-04 | 0.2 | <0.001 | 0.956 |
| dgA-11_gut_group | 1.90E-05 | 6.00E-03 | 0.00E+00 | 6.80E-03 | 0.355 | <0.001 | 0.099 |
| Lachnospiraceae_UCG-008 | 5.20E-03 | 9.20E-04 | 6.10E-03 | 1.10E-03 | 0.186 | <0.001 | 0.848 |
| Mogibacterium | 3.50E-03 | 2.80E-03 | 3.10E-03 | 2.90E-03 | 0.67 | 0.191 | 0.631 |
| **Prevotellaceae_NK3B31_group** | **7.20E-03** | **3.80E-05** | **5.30E-03** | **0.00E+00** | **<0.001** | **<0.001** | **<0.05** |
| Ruminococcaceae_UCG-009 | 2.30E-04 | 5.70E-03 | 2.20E-04 | 5.00E-03 | 0.251 | <0.001 | 0.543 |
| Anaerotruncus | 2.20E-03 | 3.20E-03 | 2.20E-03 | 3.70E-03 | 0.304 | <0.001 | 0.362 |
| Roseburia | 3.50E-03 | 1.90E-03 | 3.40E-03 | 2.60E-03 | 0.256 | <0.001 | 0.179 |
| Alloprevotella | 1.90E-04 | 5.10E-03 | 2.40E-04 | 4.70E-03 | 0.875 | <0.001 | 0.471 |
| Marvinbryantia | 3.80E-03 | 1.70E-03 | 3.50E-03 | 1.70E-03 | 0.552 | <0.001 | 0.941 |
| Cellulosilyticum | 2.10E-05 | 4.10E-03 | 1.60E-05 | 5.40E-03 | 0.145 | <0.001 | 0.132 |
| Anaeroplasma | 4.60E-03 | 9.60E-04 | 4.00E-03 | 9.20E-04 | 0.351 | <0.001 | 0.713 |
| Pseudobutyrivibrio | 4.40E-03 | 7.10E-04 | 4.30E-03 | 8.70E-04 | 0.758 | <0.001 | 0.632 |
| **unclassified_Bacteroidales_UCG-001** | **4.00E-03** | **1.80E-04** | **5.60E-03** | **4.10E-04** | **<0.001** | **<0.001** | **0.575** |
| **Anaerovorax** | **3.90E-03** | **8.80E-04** | **4.70E-03** | **6.70E-04** | **0.561** | **<0.001** | **<0.05** |
| **Ruminococcus_gauvreauii_group** | **4.30E-03** | **1.50E-03** | **3.00E-03** | **1.20E-03** | **<0.01** | **<0.001** | **0.18** |
| **Clostridium_sensu_stricto_1** | **2.20E-05** | **3.80E-03** | **4.10E-05** | **5.40E-03** | **<0.05** | **<0.001** | **0.083** |
| Phascolarctobacterium | 4.20E-05 | 4.80E-03 | 3.60E-05 | 4.40E-03 | 0.503 | <0.001 | 0.582 |
| unclassified_Peptostreptococcaceae | 0.00E+00 | 4.30E-03 | 0.00E+00 | 4.80E-03 | 0.522 | <0.001 | 0.522 |
| Lachnoclostridium_10 | 4.10E-03 | 5.50E-04 | 4.30E-03 | 8.10E-04 | 0.205 | <0.001 | 0.692 |
| Thalassospira | 2.50E-03 | 2.30E-03 | 2.00E-03 | 2.60E-03 | 0.947 | 0.53 | 0.303 |
| Olsenella | 3.40E-03 | 1.70E-03 | 2.60E-03 | 1.80E-03 | 0.511 | <0.01 | 0.358 |
| unclassified_Anaerolineaceae | 4.30E-03 | 2.70E-04 | 4.70E-03 | 3.50E-04 | 0.29 | <0.001 | 0.853 |
| Ruminococcaceae_UCG-002 | 1.20E-03 | 3.10E-03 | 1.50E-03 | 3.10E-03 | 0.514 | <0.001 | 0.53 |
| Eubacterium_nodatum_group | 2.40E-03 | 1.80E-03 | 2.50E-03 | 1.90E-03 | 0.671 | <0.01 | 0.934 |
| Anaerorhabdus_furcosa_group | 2.10E-03 | 2.00E-03 | 2.20E-03 | 2.20E-03 | 0.736 | 0.876 | 0.917 |
| Papillibacter | 4.00E-03 | 2.70E-04 | 4.00E-03 | 3.80E-04 | 0.284 | <0.001 | 0.405 |
| Eubacterium_hallii_group | 2.70E-03 | 1.20E-03 | 2.70E-03 | 1.80E-03 | 0.306 | <0.001 | 0.245 |
| unclassified_Porphyromonadaceae | 8.50E-05 | 3.80E-03 | 4.50E-05 | 3.60E-03 | 0.348 | <0.001 | 0.603 |
| Oscillibacter | 0.00E+00 | 3.70E-03 | 0.00E+00 | 3.70E-03 | 0.927 | <0.001 | 0.927 |
| Coprococcus_3 | 2.00E-05 | 4.10E-03 | 2.10E-05 | 3.30E-03 | 0.1 | <0.001 | 0.098 |
| **unclassified_NB1-n** | **7.00E-04** | **2.20E-03** | **1.10E-03** | **3.40E-03** | **<0.01** | **<0.001** | **0.35** |
| Lachnospiraceae_FCS020_group | 3.00E-03 | 6.70E-04 | 3.60E-03 | 6.50E-04 | 0.243 | <0.001 | 0.244 |
| unclassified_p-2534-18B5_gut_group | 3.70E-05 | 2.70E-03 | 2.00E-05 | 3.90E-03 | 0.72 | <0.001 | 0.655 |
| unclassified_Proteobacteria | 1.70E-03 | 1.70E-03 | 1.70E-03 | 2.00E-03 | 0.505 | 0.501 | 0.431 |
| unclassified_Family_XIII | 1.30E-03 | 1.90E-03 | 1.40E-03 | 2.30E-03 | 0.362 | <0.001 | 0.499 |
| probable_genus_10 | 3.60E-03 | 0.00E+00 | 3.60E-03 | 0.00E+00 | 0.853 | <0.001 | 0.853 |
| Romboutsia | 4.30E-05 | 3.00E-03 | 2.10E-05 | 3.50E-03 | 0.476 | <0.001 | 0.13 |
| **Sutterella** | **6.00E-04** | **2.20E-03** | **1.40E-03** | **2.40E-03** | **<0.05** | **<0.001** | **0.064** |
| **Lachnospiraceae_ND3007_group** | **2.90E-03** | **3.90E-05** | **3.90E-03** | **1.80E-05** | **0.126** | **<0.001** | **<0.05** |
| unclassified_Alphaproteobacteria | 2.30E-03 | 7.40E-04 | 2.40E-03 | 1.00E-03 | 0.435 | <0.001 | 0.658 |
| Solobacterium | 1.90E-03 | 1.30E-03 | 1.50E-03 | 1.70E-03 | 0.853 | 0.323 | 0.086 |
| unclassified_Candidate_division_SR4 | 2.70E-03 | 7.50E-05 | 3.70E-03 | 5.30E-05 | 0.234 | <0.001 | 0.095 |
| Coprococcus_1 | 1.10E-03 | 1.60E-03 | 1.40E-03 | 2.00E-03 | 0.08 | <0.001 | 0.871 |
| Lachnospiraceae_UCG-006 | 2.90E-03 | 1.00E-04 | 3.10E-03 | 1.20E-04 | 0.728 | <0.001 | 0.651 |
| Eubacterium_brachy_group | 7.80E-04 | 2.20E-03 | 4.70E-04 | 2.10E-03 | 0.094 | <0.001 | 0.136 |
| **unclassified_Clostridia** | **6.50E-04** | **2.60E-03** | **3.20E-04** | **1.90E-03** | **<0.01** | **<0.001** | **0.791** |
| Ruminobacter | 1.30E-03 | 1.00E-03 | 2.30E-03 | 7.40E-04 | 0.528 | <0.01 | 0.12 |
