# Supplementary material for: The Structural and Functional Capacity of Ruminal and Cecal Microbiota in Growing Cattle Was Unaffected by Dietary Supplementation of Linseed Oil and Nitrate
Source: Front Microbiol. 2017 May 24;8:937. doi: 10.3389/fmicb.2017.00937 (PMC5442214; doi:10.3389/fmicb.2017.00937)
Supplement: Supplementary file 6 [file Table6.docx]

Table S6 Comparison of PICRUSt metabolic pathway prediction in rumen and caecum contents of control- (CTL) and linseed and nitrates- (LINNIT) fed growing bulls

|  | CTL | | LINNIT | |  | Effect | | |
| --- | --- | --- | --- | --- | --- | --- | --- | --- |
|  | rumen | caecum | rumen | caecum | SEM | Trt | Compart | Trt*Compart |
| **Cellular processes and signalling (%)** | 10.43 | 10.68 | 10.48 | 10.84 | 2.59 | 0.19 | <0.001 | 0.47 |
| **Carbohydrate metabolism (%)** | 10.14 | 10.13 | 10.09 | 10.12 | 0.4462 | 0.26 | 0.73 | 0.44 |
| **Amino acid metabolism (%)** | 8.07 | 7.94 | 8.06 | 7.89 | 0.272 | 0.34 | <0.001 | 0.56 |
| **Replication and repair (%)** | 7.89 | 7.79 | 7.86 | 7.73 | 0.377 | 0.29 | <0.05 | 0.61 |
| **Translation (%)** | 6.63 | 6.51 | 6.61 | 6.44 | 0.423 | 0.25 | <0.001 | 0.55 |
| **Energy metabolism (%)** | 6.38 | 6.42 | 6.35 | 6.35 | 0.5672 | 0.23 | 0.60 | 0.50 |
| **Membrane transport (%)** | 5.44 | 5.50 | 5.46 | 5.60 | 1.461 | 0.23 | <0.05 | 0.37 |
| **Metabolism of cofactors and vitamins (%)** | 4.18 | 4.17 | 4.20 | 4.16 | 0.1576 | 0.99 | 0.32 | 0.53 |
| **Nucleotide metabolism (%)** | 4.02 | 3.96 | 4.01 | 3.92 | 0.234 | 0.26 | <0.001 | 0.39 |
| **Folding, sorting and degradation (%)** | 3.03 | 3.03 | 3.04 | 3.02 | 0.05273 | 0.97 | 0.85 | 0.61 |
| **Lipids metabolism (%)** | 2.59 | 2.56 | 2.60 | 2.58 | 0.0182 | 0.17 | <0.05 | 0.64 |
| **Glycan biosynthesis and metabolism (%)** | 2.17 | 2.22 | 2.18 | 2.18 | 0.4191 | 0.47 | 0.25 | 0.31 |
| **Xenobiotics biodegradation and metabolism (%)** | 1.62 | 1.60 | 1.62 | 1.62 | 0.05574 | 0.21 | <0.1 | 0.27 |
| **Metabolism of terpenoids and polyketides (%)** | 1.54 | 1.51 | 1.53 | 1.50 | 0.0016 | 0.55 | <0.1 | 0.92 |
| **Transcription (%)** | 1.14 | 1.16 | 1.14 | 1.17 | 0.001 | 0.47 | <0.001 | 0.84 |
| **Biosynthesis of other secondary metabolites (%)** | 0.91 | 0.90 | 0.91 | 0.89 | 0.01354 | 0.66 | <0.01 | 0.37 |
| **Others (%)** | 23.77 | 23.91 | 23.82 | 24.10 | 3.5 | 0.44 | 0.17 | 0.66 |
